# Supplementary material for: Generalization of procedural motor sequence learning after a single practice trial
Source: NPJ Sci Learn. 2023 Oct 6;8:45. doi: 10.1038/s41539-023-00194-7 (PMC10558563; doi:10.1038/s41539-023-00194-7)
Supplement: Supplementary file 1 — Supplemental Information [file 41539_2023_194_MOESM1_ESM.docx]

Generalization of procedural motor sequence learning after a single practice trial: Supplementary information

B.P. Johnson^1,2^, I. Iturrate^1,3^, R.Y. Fakhreddine^1,4^, M. Bönstrup^5^, E.R. Buch^1*^, E.M. Robertson^6^, and L.G. Cohen^1*^

*Corresponding authors

^1^ Human Cortical Physiology and Neurorehabilitation Section, NINDS, NIH; ^2^ Washington University in St Louis; ^3^ Amazon EU; ^4^ UT Austin; ^5^ University of Leipzig Germany; ^6^ Center for Cognitive Neuroimaging, University of Glasgow, Scotland, UK

L.G. Cohen: cohenl@ninds.nih.gov

E.R. Buch: ethan.buch@nih.gov

**Supplementary Discussion**

*Experiment 1:*

There was no significant between group difference in initial performance on skill A (F _(3, 547)_ = 0.712, p = 0.545). Increasing duration of practice in skill A did not improve initial performance in skill B (Correct keypress/s: 1T: 2.948 ± 1.193; 2T: 2.731 ± 1.158; 5T: 3.064 ± 1.375 ;12T: 2.660 ± 1.452; Kruskal-Wallis Test: X^2^(3, 547) = 3.229, p = 0.358; **Fig 1b and 1c**). This performance at the onset of skill B was statistically similar between-groups (Bayes Factor = 0.002).

The 12-trial practice group (12T) reached plateau by trial 11, consistent with previous work (**Fig 1b**) (1). All groups except 12T demonstrated positive ${Gen}_{B_{0}-A_{0}}$ (1T: 0.622 ± 1.104; 2T: 0.496 ± 1.384; 5T: 0.737 ± 1.447; 12T: 0.184 ± 1.625; one-sample *t*-test: *t* = [4.256 6.640], *p* < 0.001 for all groups except 12T: *t* = 1.325, *p* = 0.187, **Supplementary Fig 2a**). The magnitude of positive generalization did not differ between groups (Kruskal-Wallis Test: *X^2^*_(3, 547)_ = 7.006, *p* = 0.072) and was statistically similar between-groups (Bayes Factor = 0.053).

The learning rate ($\kappa$) provides information on the extent to which practice of the first skill A influenced how quickly performance improvement occurred for the new skill B (31). Here, we found no between-group differences in the learning rate of skill B ($\kappa_{B}$; One-way ANOVA: *F*_(3, 533)_ = 0.822, *p* = 0.482; $\kappa_{B}$: 1T: 0.351 ± 0.477 ;2T: 0.438 ± 0.521 ;5T: 0.427 ± 0.488 ;12T: 0.402 ± 0.504; **Supplementary Fig 2b**). We found no differences in learning rates of both skills as a function of practice duration (**Supplementary Fig 2b**). The learning rate of skill B was statistically similar between-groups (Bayes Factor = 0.000).

*Experiment 2:*

There was no statistically significant between group difference in initial performance on skill A (F _(4, 790)_ = 0.994, p = 0.410). Inter-skill rest intervals did not modify one-trial generalization for $\mathrm{Gen}_{B_{0}-A_{0}}$ (One-way ANOVA: *F*_(4, 794)_ = 0.334, *p* = 0.855; **Supplementary Fig 4a**) or $\mathrm{Gen}_{B_{0}-A_{f}}$ (Kruskal-Wallis test: *X^2^*_(4, 794)_ = 3.793, *p* = 0.435; **Fig 2c**) and generalization was statistically similar between-groups ($\mathrm{Gen}_{B_{0}-A_{0}}:$Bayes Factor = 0.000; $\mathrm{Gen}_{B_{0}-A_{f}}:$Bayes Factor = 0.038 Finally, no statistically significant between-group differences were found for the learning rate of skill B ($\kappa_{B};$One-way ANOVA: *F*_(4, 733_) = 0.922, *p* = 0.451; **Supplementary Fig 4b**). The learning rate of skill B was statistically similar between-groups (Bayes Factor = 0.000).

*Experiment 3:*

There was no statistically significant between group difference in initial performance on skill A (F _(3, 533)_ = 1.495, p = 0.215). Similar between-group generalization differences were observed for ${Gen}_{B_{0}-A_{f}}$ (One-way ANOVA: *F*_(3, 533)_ = 7.550, *p* < 0.001; Correct keypress/s change: PARSING: 0.296 ± 1.510; TRANSITION: 0.027 ± 1.225; O+T: -0.048 ± 1.288; SAME_A_: 0.637 ± 1.188; **Fig 3d**). SAME_A_ showed significantly greater ${Gen}_{B_{0}-A_{f}}$ than TRANSITION (*p* = 0.001) and O+T (*p* < 0.001) (both FWE-corrected), but not compared with PARSING (*p* = 0.192). Both SAME_A_ and PARSING showed positive ${Gen}_{B_{0}-A_{0}}$ while TRANSITION and O+T did not (One-sample *t*-tests: SAME_A_, *t* = 6.299, *p* < 0.001; PARSING, *t* = 2.280, *p* = 0.024; TRANSITION, *t* = 0.259, *p* = 0.796; O+T, *t* = -0.397, *p* = 0.692; **Supplementary Fig 6a**). Lastly, there were no between-group differences in skill B learning rates ($\kappa_{B};$Kruskal-Wallis test: *X^2^*_(3, 529)_ = 0.752, *p* = 0.861; **Supplementary Fig 6b**). The learning rate of skill B was statistically similar between-groups (Bayes Factor = 0.053).

**Supplementary Figures**

Supplementary Fig 1.


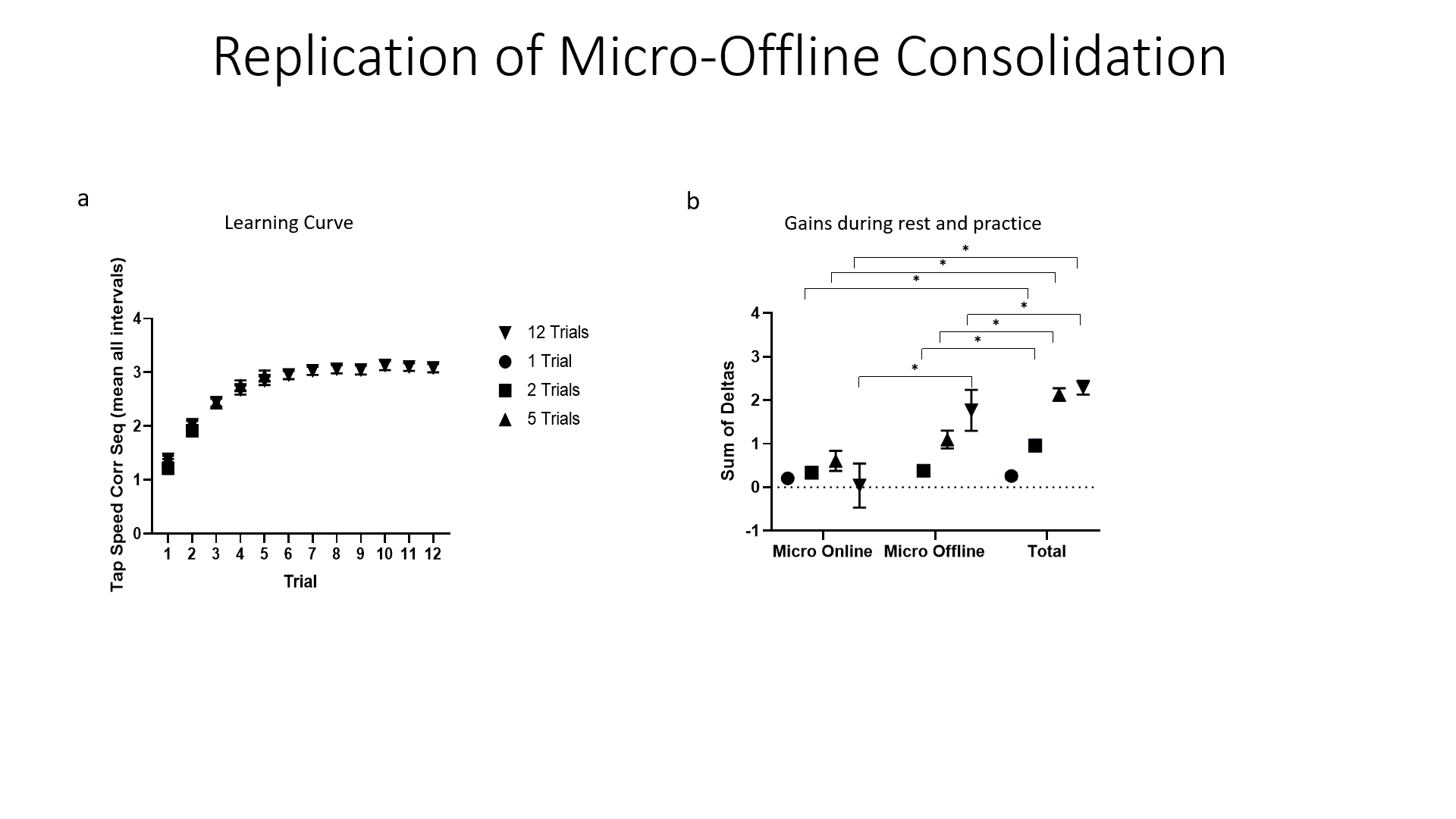


***Supplementary Fig 1.*** *Learning curves (****a****), micro-online and micro-offline gains during rest and practice (****b****) in all groups, Experiment 1. Note the overlapping, similar learning curves in all groups that practiced Skill A for different number of trials (****a****). Importantly, note that for all groups, total learning was virtually fully accounted for by micro-offline gains, consistent with previous work (****b****) (1,2).* *Error bars indicate standard error of the mean. * p < 0.05, where* ** between plots indicate significant repeated-measures ANOVA (pairwise comparison) within-group differences.*

Supplementary Fig 2.


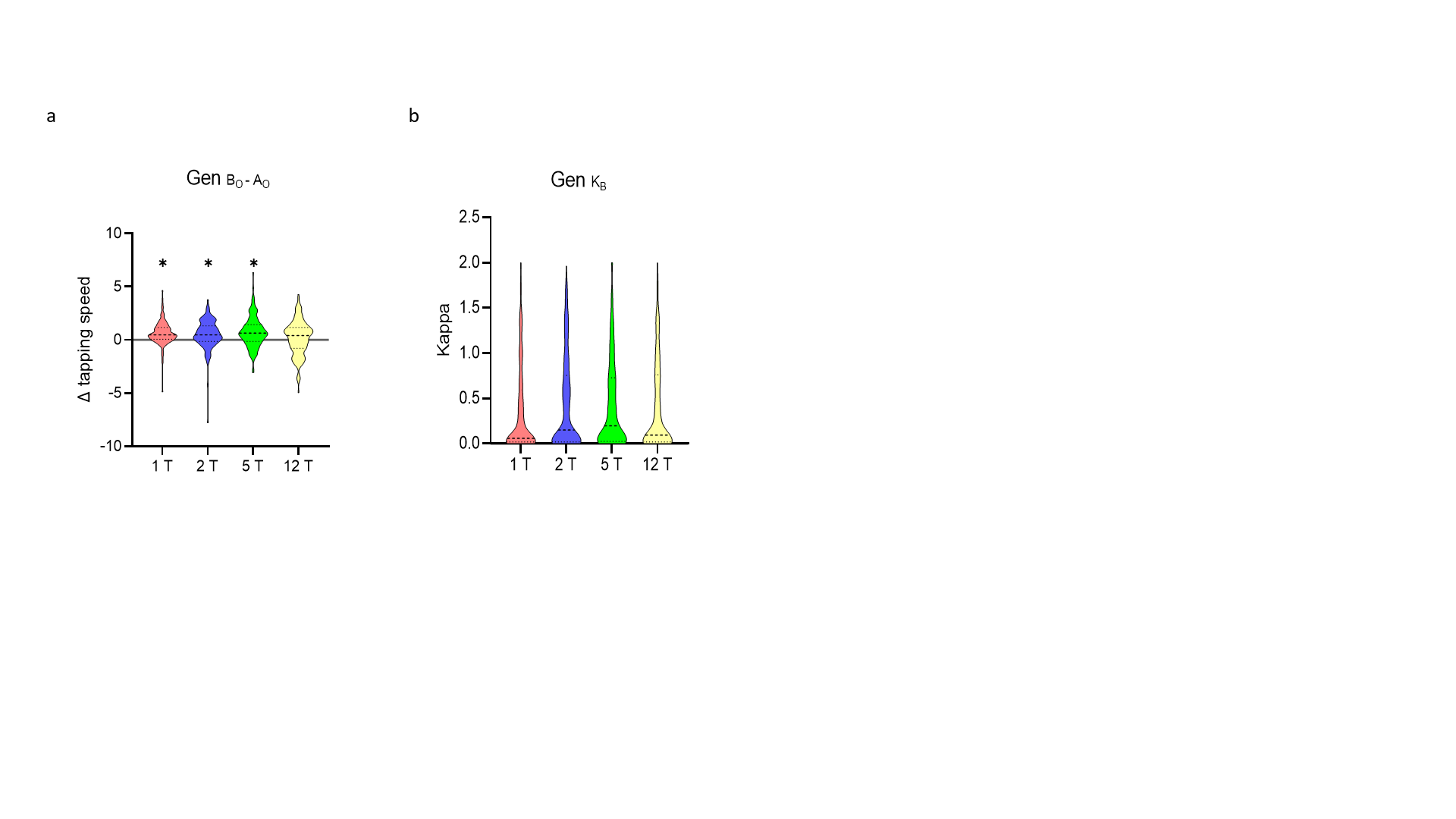


***Supplementary Figure 2.*** ***Influence of length of training of skill A on generalization to skill B during early learning****.* ***(a)*** *Change in skill from the onset of skill A to the onset of skill B (i.e.,* ${Gen}_{B_{0}-A_{0}}$*). All groups demonstrated positive* ${Gen}_{B_{0}-A_{0}}$*but note that this improvement was significant for all groups except the 12 trials group.* ***(b).*** *Growth rate in performance across the five trials of skill B (i.e.,* $\kappa_{B}$*). There were no between-group differences in the growth rate of skill B. * p < 0.05, where * over individual group plots indicate significant one-sample t-test within-group differences between B_0_ and A_0_.*

Supplementary Fig 3.


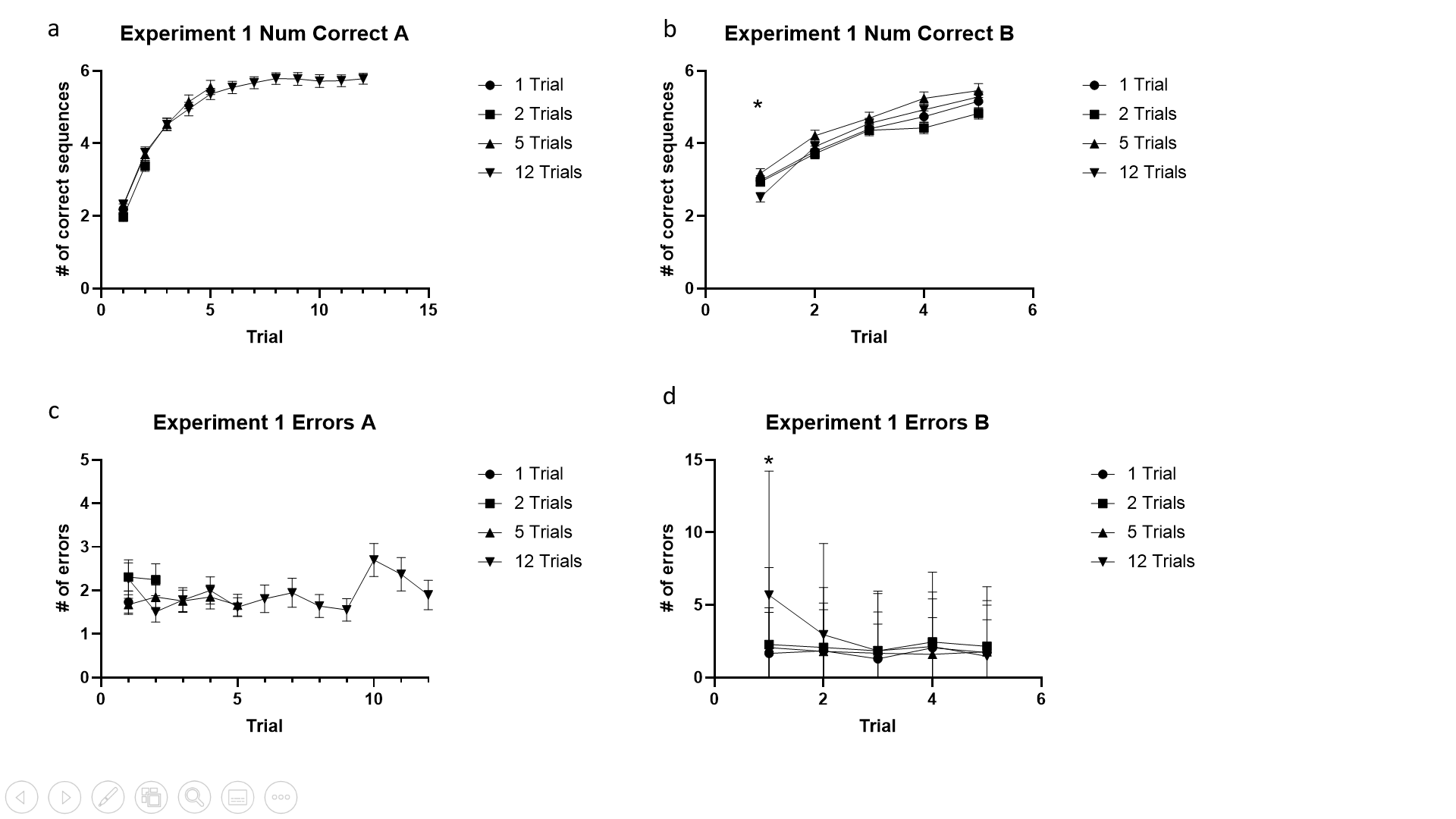


***Supplementary Fig 3.*** *Number of correct sequences for skills A and B (****a*** *and* ***b****) and accuracy (number of errors,* ***c*** *and* ***d****) in Experiment 1. Note the similar performance and accuracy in all four experimental groups. The 12 trials group had significantly fewer correct sequences during the first trial of sequence B (One-way ANOVA: F (3, 550) = 4.916, p = 0.002) compared to the 1 trial (p = 0.046) and 5 trials (p = 0.001) groups (****b****). In addition, the 12 trials group had significantly more errors during the 1^st^ trial of Sequence B (Kruskal Wallis test: X^2^ = 44.056; p < 0.001) than all other groups (post-hoc tests corrected for family-wise error: p ≤ 0.002 for all;* ***d****). Error bars indicate standard error of the mean.*

Supplementary Fig 4.


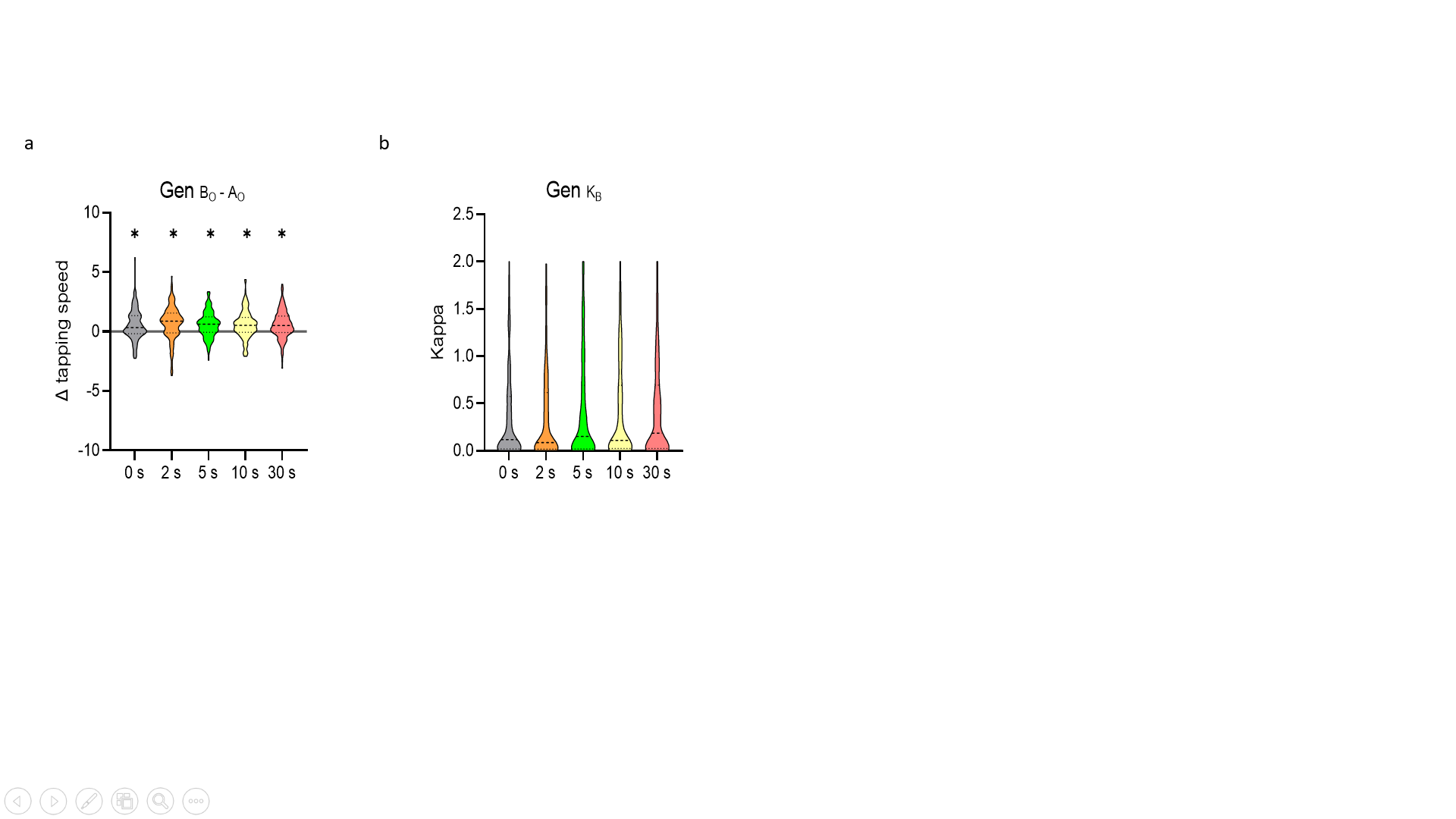


**Supplementary Figure 4. Varying rest interval durations does not impact rapid generalization during early learning.** **(a)** Change in skill from the onset of skill A to the onset of skill B (i.e., ${Gen}_{B_{0}-A_{0}}$). All groups demonstrated positive ${Gen}_{B_{0}-A_{0}}$, while there were no between-group differences. **(b)** Growth rate in performance across the five trials of skill B (i.e., $\kappa_{B}$). There were no between-group differences in the growth rate of skill B. * p < 0.05, where * over individual group plots indicate significant one-sample t-test within-group differences between B_0_ and A_0_.

Supplementary Fig 5.

Number of correct sequences (**a** and **b**) and accuracy (number of errors, **c** and **d**) in Experiment 2. Note the similar performance and accuracy in all five experimental
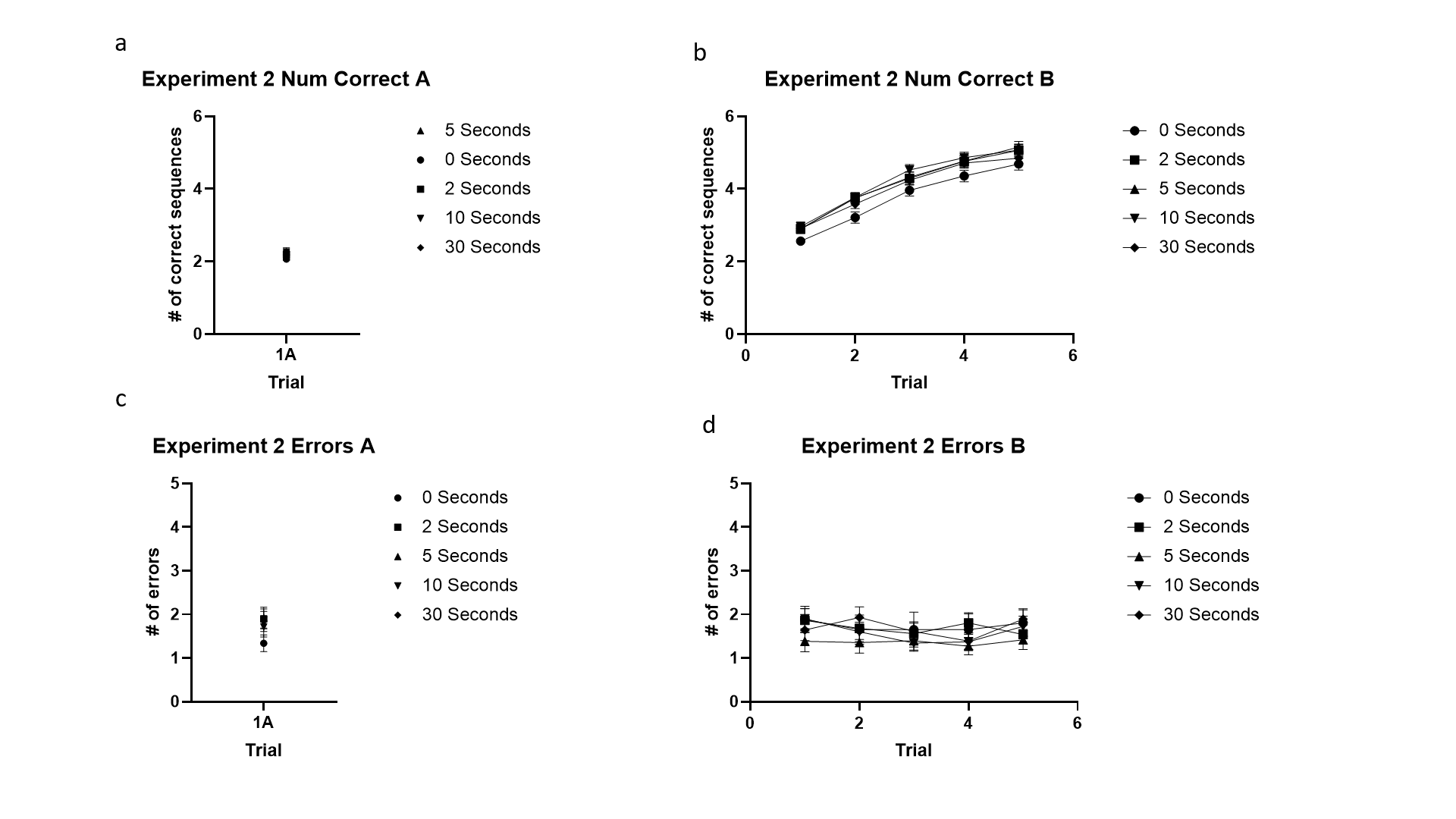


*

groups except for the lower number of correct sequences when the interval between skills was 0sec (One-way ANOVA: *F* = 2.892; *p* = 0.022) (**d**). Error bars indicate standard error of the mean.

Supplementary Fig 6.


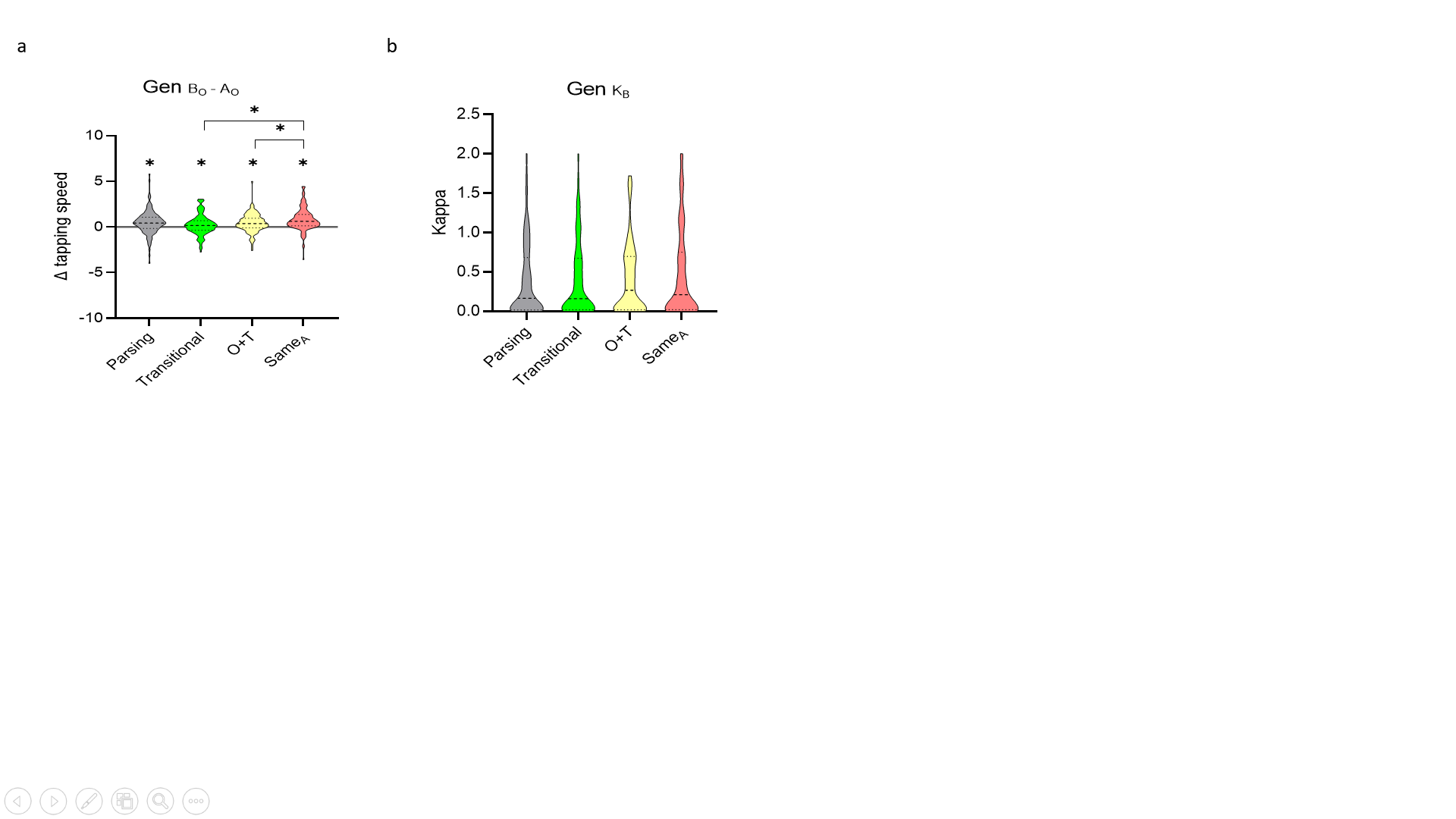


*Supplementary Figure 6.* ***Content of generalization during early skill learning. (a)*** *Change in skill from the onset of skill A to the onset of skill B (i.e.,* ${Gen}_{B_{0}-A_{0}}$*). All groups demonstrated* ${Gen}_{B_{0}-A_{0}}$*, though between-group differences were evident with clear superiority of the SAME_A_ over the TRANSITION and the O+T groups.* ***(b)*** *Growth rate in performance across the five trials of skill B (i.e.,* $\kappa_{B}$*). There were no between-group differences in the growth rate of skill B.* * *p* < 0.05, where * over individual group plots indicate significant one-sample t-test within-group differences between B_0_ and A_0_.

Supplementary Fig 7.


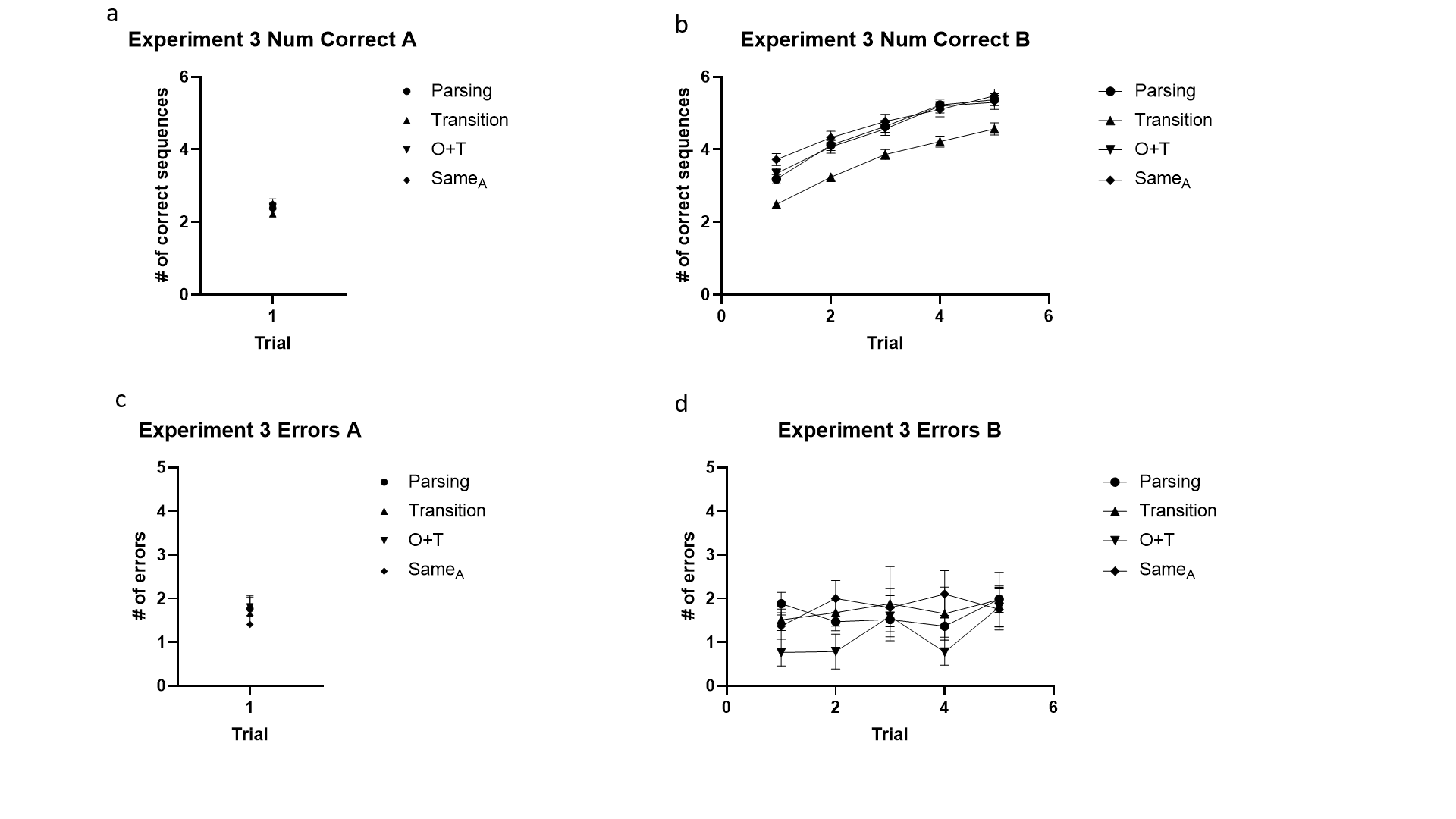


*

*

*

*

*

*

*Supplementary Figure 7. Number of correct sequences (****a*** *and* ***b****) and accuracy (number of errors,* ***c*** *and* ***d****) in Experiment 3. The TRANSITION group had a significantly lower number of correct skill B sequences than the other groups in all trials (Kruskal Wallis test for trials 1-4: p ≤ 0.001 for all; One-way ANOVA for trial 5: p = 0.001,* ***b****), but had a comparable learning curve and overall number of errors (****d****). Error bars indicate standard error of the mean.*

Supplementary Fig 8.


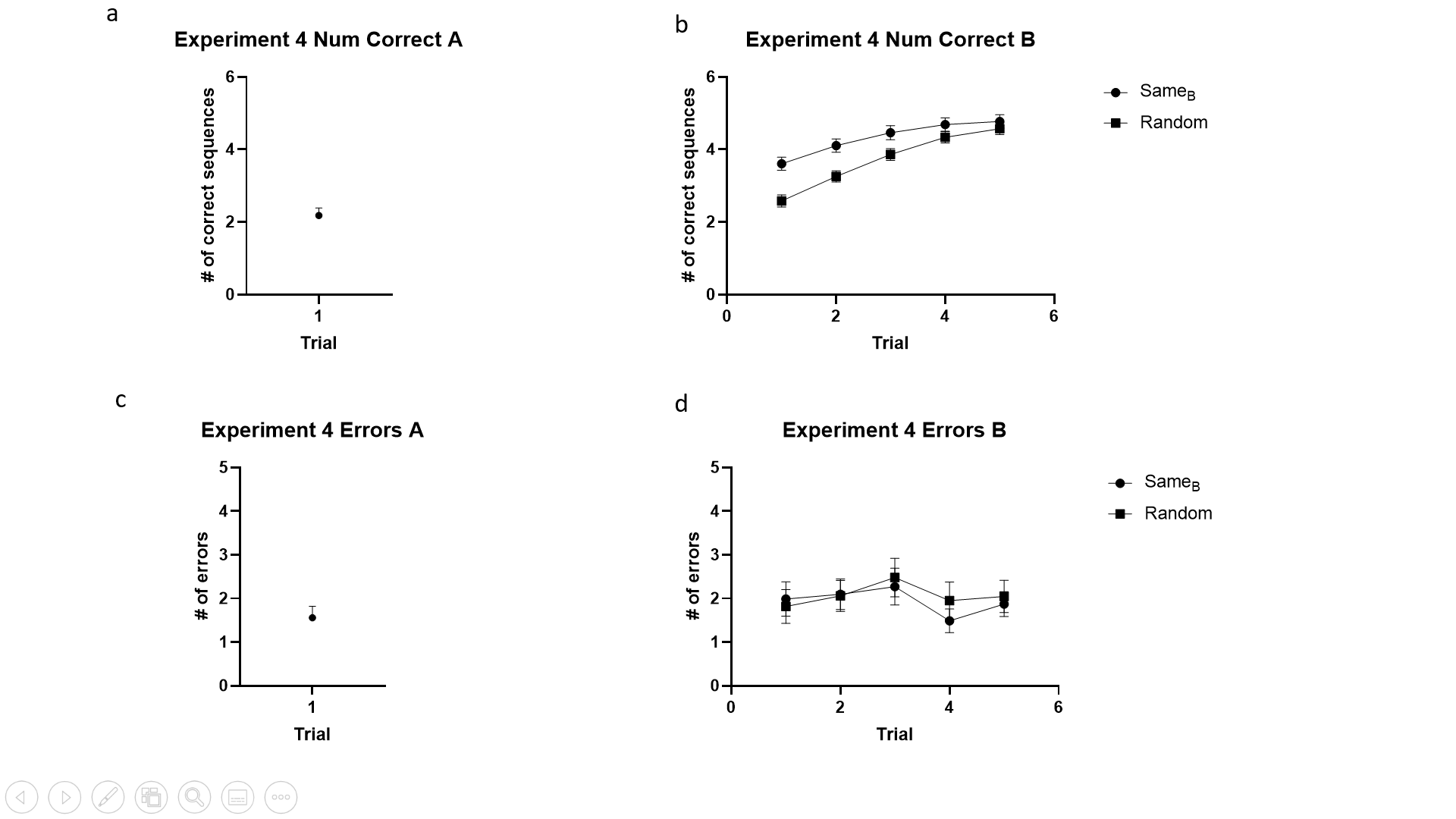


*

*

*

*

***Supplementary Fig 8.*** *Number of correct sequences (****a*** *and* ***b****) and accuracy (number of errors,* ***c*** *and* ***d****) in Experiment 4. Note the larger number of correct sequences during trials 1-4 (independent t-tests p < 0.05 for trials 1-4) when subjects performed skill B twice (****b****). Error bars indicate standard error of the mean.*

Supplementary Fig 9.


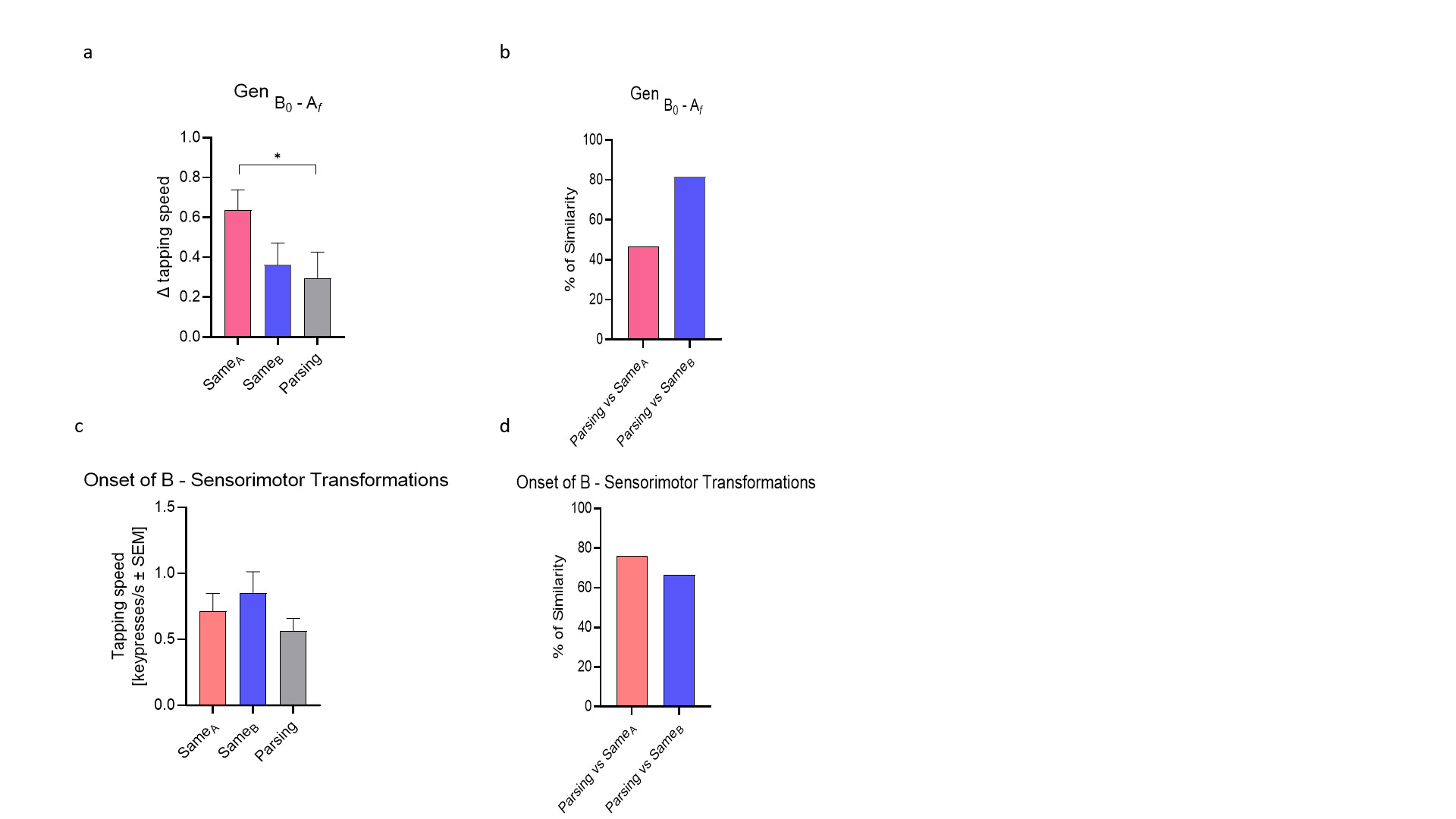


***Supplementary Fig 9.*** ${Gen}_{B_{0}-A_{f}}$ *(i.e., micro-offline generalization) in the Parsing group (0.297 ± 0.130 correct keypresses/s change) was 46.578% and 81.736% of that in the SAME_A_ and SAME_B_ (0.637 ± 0.101 and 0.363 ± 0.109 correct keypresses/s change, respectively, independent-samples t-test: t (_271_) = -2.074, p = 0.039 and t (_1, 238_) = 0.379, p = 0.705) suggesting that the magnitude of micro-offline consolidation and generalization differ depending on skill structure (****a*** *and* ***b****). Furthermore, the onset of B in the PARSING group (2.967 ± 1.104 correct keypresses/s) was 76.297% and 66.748% of that in the SAME_A_ and SAME_B_ groups (3.136 ± 1.602 and 3.238 ± 1.612 correct keypresses/s, respectively) after subtracting the influence of sensorimotor transformations from all three groups (i.e., onset of B for the RANDOM group, 2.423 ± 1.310 correct keypresses/s) (****c*** *and* ***d****). Error bars indicate standard error of the mean.*

**Supplementary Tables**

Supplementary table 1. Demographic information for Experiment 1

|  | | Trials of Seq A | | | |
| --- | --- | --- | --- | --- | --- |
|  |  | 1 Trial | 2 Trials | 5 Trials | 12 Trials |
|  |  | Count | Count | Count | Count |
| Mean Age (SD) |  | 38.1 (11.2) | 35.4 (9.6) | 37.3 (11.4) | 35.9 (10.6) |
| Gender | Women | 55 | 48 | 53 | 61 |
|  | Men | 84 | 92 | 80 | 74 |
|  | Other | 0 | 1 | 1 | 2 |
| Ethnicity | Hispanic or Latino | 8 | 17 | 13 | 10 |
|  | Not Hispanic or Latino | 130 | 122 | 119 | 127 |
|  | Unknown/ Not Reported Ethnicity | 1 | 2 | 2 | 0 |
| Race | American Indian/Alaska Native | 1 | 1 | 0 | 0 |
|  | Asian | 0 | 0 | 0 | 1 |
|  | Native Hawaiin or Other Pacific Islander | 6 | 6 | 13 | 10 |
|  | Black or African American | 14 | 12 | 14 | 16 |
|  | White | 116 | 119 | 104 | 104 |
|  | More Than One Race | 2 | 2 | 2 | 5 |
|  | Unknown or Not Reported | 0 | 1 | 1 | 1 |
| Instrument | Does not play musical instrument | 98 | 95 | 85 | 106 |
|  | <2h/week | 25 | 25 | 26 | 16 |
|  | >2h/week | 16 | 21 | 23 | 15 |

Supplementary table 2. Demographic information for Experiment 2

|  | | Inter Skill Rest | | | | |
| --- | --- | --- | --- | --- | --- | --- |
|  |  | 0 s | 2 s | 5 s | 10 s | 30 s |
|  |  | Count | Count | Count | Count | Count |
| Mean Age (SD) |  | 36.7 (10.5) | 36.3 (10.2) | 37.4 (11.6) | 36.5 (11.2) | 37.0 (11.6) |
| Gender | Women | 72 | 70 | 68 | 74 | 66 |
|  | Men | 84 | 84 | 100 | 80 | 96 |
|  | Other | 0 | 1 | 0 | 0 | 0 |
| Ethnicity | Hispanic or Latino | 14 | 15 | 18 | 13 | 23 |
|  | Not Hispanic or Latino | 141 | 139 | 149 | 141 | 139 |
|  | Unknown/ Not Reported Ethnicity | 1 | 0 | 1 | 1 | 0 |
| Race | American Indian/Alaska Native | 0 | 0 | 5 | 0 | 0 |
|  | Asian | 0 | 0 | 0 | 0 | 1 |
|  | Native Hawaiin or Other Pacific Islander | 9 | 15 | 16 | 20 | 20 |
|  | Black or African American | 11 | 13 | 15 | 10 | 16 |
|  | White | 135 | 119 | 127 | 117 | 123 |
|  | More Than One Race | 0 | 6 | 7 | 7 | 2 |
|  | Unknown or Not Reported | 1 | 1 | 2 | 1 | 0 |
| Instrument | Does not play musical instrument | 109 | 110 | 126 | 112 | 105 |
|  | <2h/week | 22 | 28 | 27 | 22 | 32 |
|  | >2h/week | 25 | 16 | 15 | 21 | 25 |

Supplementary table 3. Demographic information for Experiment 3

|  | | Sequence Similarity | | | | |
| --- | --- | --- | --- | --- | --- | --- |
|  |  | Parsing | Transition | O+T | Same_A_ |  |
|  |  | Count | Count | Count | Count |  |
| Mean Age (SD) |  | 36.9 (12.2) | 37.1 (11.4) | 37.3 (11.3) | 39.4 (12.2) |  |
| Gender | Women | 65 | 67 | 70 | 66 |  |
|  | Men | 70 | 64 | 61 | 71 |  |
|  | Other | 0 | 2 | 0 | 1 |  |
| Ethnicity | Hispanic or Latino | 15 | 5 | 12 | 8 |  |
|  | Not Hispanic or Latino | 117 | 125 | 119 | 129 |  |
|  | Unknown/ Not Reported Ethnicity | 3 | 3 | 0 | 1 |  |
| Race | American Indian/Alaska Native | 2 | 0 | 2 | 3 |  |
|  | Asian | 0 | 0 | 0 | 0 |  |
|  | Native Hawaiin or Other Pacific Islander | 16 | 11 | 13 | 12 |  |
|  | Black or African American | 8 | 8 | 11 | 11 |  |
|  | White | 103 | 103 | 104 | 106 |  |
|  | More Than One Race | 3 | 7 | 1 | 4 |  |
|  | Unknown or Not Reported | 3 | 4 | 0 | 2 |  |
| Instrument | Does not play musical instrument | 101 | 103 | 96 | 103 |  |
|  | <2h/week | 23 | 19 | 24 | 19 |  |
|  | >2h/week | 11 | 11 | 11 | 16 |  |

Supplementary table 4. Demographic information for Experiment 4

|  | |  |  |
| --- | --- | --- | --- |
|  |  | Same_B_ | Random |
|  |  | Count | Count |
| Mean Age (SD) |  | 37.7 (9.0) | 36.4 (8.2) |
| Gender | Women | 44 | 37 |
|  | Men | 61 | 70 |
|  | Other | 0 | 0 |
| Ethnicity | Hispanic or Latino | 26 | 19 |
|  | Not Hispanic or Latino | 79 | 87 |
|  | Unknown/ Not Reported Ethnicity | 0 | 1 |
| Race | American Indian/Alaska Native | 2 | 0 |
|  | Asian | 0 | 0 |
|  | Native Hawaiin or Other Pacific Islander | 8 | 2 |
|  | Black or African American | 22 | 9 |
|  | White | 72 | 95 |
|  | More Than One Race | 1 | 1 |
|  | Unknown or Not Reported | 0 | 0 |
| Instrument | Does not play musical instrument | 38 | 42 |
|  | <2h/week | 40 | 24 |
|  | >2h/week | 27 | 41 |
