# Supplementary material for: Generalization of procedural motor sequence learning after a single practice trial
Source: NPJ Sci Learn. 2023 Oct 6;8:45. doi: 10.1038/s41539-023-00194-7 (PMC10558563; doi:10.1038/s41539-023-00194-7)
Supplement: Supplementary file 2 — Reporting summary [file 41539_2023_194_MOESM2_ESM.pdf]

nature portfolio

Corresponding author(s): Leonardo G. Cohen; Ethan R. Buch

Last updated by author(s): Mar 21, 2023

Reporting Summary

Nature Portfolio wishes to improve the reproducibility of the work that we publish. This form provides structure for consistency and transparency in reporting. For further information on Nature Portfolio policies, see our Editorial Policies and the Editorial Policy Checklist.

Statistics

For all statistical analyses, confirm that the following items are present in the figure legend, table legend, main text, or Methods section.

n/a	Confirmed	
☐	☒	The exact sample size (*n*) for each experimental group/condition, given as a discrete number and unit of measurement
☐	☒	A statement on whether measurements were taken from distinct samples or whether the same sample was measured repeatedly
☐	☒	The statistical test(s) used AND whether they are one- or two-sided<br>*Only common tests should be described solely by name; describe more complex techniques in the Methods section.*
☐	☒	A description of all covariates tested
☐	☒	A description of any assumptions or corrections, such as tests of normality and adjustment for multiple comparisons
☐	☒	A full description of the statistical parameters including central tendency (e.g. means) or other basic estimates (e.g. regression coefficient) AND variation (e.g. standard deviation) or associated estimates of uncertainty (e.g. confidence intervals)
☐	☒	For null hypothesis testing, the test statistic (e.g. *F*, *t*, *r*) with confidence intervals, effect sizes, degrees of freedom and *P* value noted<br>*Give P values as exact values whenever suitable.*
☐	☒	For Bayesian analysis, information on the choice of priors and Markov chain Monte Carlo settings
☒	☐	For hierarchical and complex designs, identification of the appropriate level for tests and full reporting of outcomes
☐	☒	Estimates of effect sizes (e.g. Cohen's *d*, Pearson's *r*), indicating how they were calculated

Our web collection on statistics for biologists contains articles on many of the points above.

Software and code

Policy information about availability of computer code

Data collection	Custom code is available upon request by contacting the corresponding authors.
Data analysis	Custom code is available upon request by contacting the corresponding authors.

For manuscripts utilizing custom algorithms or software that are central to the research but not yet described in published literature, software must be made available to editors and reviewers. We strongly encourage code deposition in a community repository (e.g. GitHub). See the Nature Portfolio guidelines for submitting code & software for further information.

Data

Policy information about availability of data

All manuscripts must include a data availability statement. This statement should provide the following information, where applicable:

- Accession codes, unique identifiers, or web links for publicly available datasets
- A description of any restrictions on data availability
- For clinical datasets or third party data, please ensure that the statement adheres to our policy

Behavioral data are available upon request by contacting the corresponding authors.

Human research participants

Policy information about studies involving human research participants and Sex and Gender in Research.

Reporting on sex and gender	Gender-based demographic information was collected via self-report, with the options of: male, female, or other. The number of participants identifying as their gender aligning with one of these three categories is reported. In total, 1171 participants identified as male, 916 identified as female, and 8 identified as other.
Population characteristics	After excluding subjects that did not adhere to the task instructions, the total sample size for all experiments in this study was N = 2,095. Experiment 1 included 551 participants (216 female, 330 male, 4 other; M ± SD age 36.658 ± 10.748, Suppl Table 1), Experiment 2 included 795 participants (350 female, 444 male, 1 other; M ± SD age 36.794 ± 11.031, Suppl Table 2), Experiment 3 included 537 participants (268 females, 266 males, 3 other; M ± SD age 37.7 ± 11.800, Suppl Table 3) and Experiment 4 included 212 participants (81 females, 131 males, 0 other; M ± SD age 37.08 ± 8.630 Suppl Table 4). Participants were randomly assigned to all groups in each experiment.
Recruitment	All participants were recruited from Amazon Mechanical Turk (MTurk) and agreed to participate via an online acknowledgement of participation, rather than an informed consent form as this study was deemed exempt from the IRB.
Ethics oversight	This study was approved by the Combined Neuroscience Institutional Review Board (IRB) of the National Institutes of Health. All participants were recruited from Amazon Mechanical Turk (MTurk) and agreed to participate via an online acknowledgement of participation, rather than an informed consent form as this study was deemed exempt from the IRB.

Note that full information on the approval of the study protocol must also be provided in the manuscript.

Field-specific reporting

Please select the one below that is the best fit for your research. If you are not sure, read the appropriate sections before making your selection.

☐ Life sciences ☒ Behavioural & social sciences ☐ Ecological, evolutionary & environmental sciences

For a reference copy of the document with all sections, see nature.com/documents/nr-reporting-summary-flat.pdf

Behavioural & social sciences study design

All studies must disclose on these points even when the disclosure is negative.

Study description	This study involved quantitative data collection of behavioral data.
Research sample	All participants were recruited from Amazon Mechanical Turk (MTurk) and agreed to participate via an online acknowledgement of participation, rather than an informed consent form as this study was deemed exempt from the IRB. Inclusion criteria were: >18 years of age, right-handedness, and living in the United States. The exclusion criterion included participation in previous sequence learning studies from our laboratory. Sample sizes were determined by conducting power analyses on pilot data previously collected on MTurk in our lab. After excluding subjects that did not adhere to the task instructions, the total sample size for all experiments in this study was N = 2,095. Experiment 1 included 551 participants (216 female, 330 male, 4 other; M ± SD age 36.658 ± 10.748, Suppl Table 1), Experiment 2 included 795 participants (350 female, 444 male, 1 other; M ± SD age 36.794 ± 11.031, Suppl Table 2), Experiment 3 included 537 participants (268 females, 266 males, 3 other; M ± SD age 37.7 ± 11.800, Suppl Table 3) and Experiment 4 included 212 participants (81 females, 131 males, 0 other; M ± SD age 37.08 ± 8.630 Suppl Table 4). Participants were randomly assigned to all groups in each experiment.
Sampling strategy	Sample sizes were determined by conducting power analyses on pilot data previously collected on MTurk in our lab. Participants were randomly assigned to all groups in each experiment.
Data collection	Participants completed behavioral studies online via their own computer keyboard, without research personnel present.
Timing	Given the step-wise nature of this study, involving multiple experiments, there were at times large gaps in time (i.e., weeks to months) between data collection. Within each experiment, there were often several days between data collection periods as the study was available for mid-day (i.e., 1000-1600 Eastern Time) data collection and small batches of data collection allowed for data quality checks. The first batch was collected on October 18, 2020 and the final batch was collected on January 21, 2022.
Data exclusions	Each participant's performance was checked for adherence to task instructions. Task assignments were deemed to not adhere to task instructions if any of the following occurred: (1) participants answered that they used their right hand to type the sequence when asked at the end which hand they used; (2) completion of only one repetition of the sequence beyond trial 1 of a given sequence; (3) keypresses were consistently different from the instructed sequences; (4) deterioration of tapping speed performance over consecutive trials after an initial increase in tapping speed performance. In total, 6,156 participants were enrolled in this study. Those who were excluded were excluded because they may have met any of the above criteria, or clicked on the study link (which we considered enrollment into the study) and never completed the study.

Non-participation	In total, 881 individuals initially clicked on the study link, thus enrolling them in the study, but never finished the online study. Given the remote nature of this study we were unable to discern the reason(s) for these occurrences.
Randomization	Participants were randomized into groups prior to enrollment via the random function in Microsoft Excel.

Reporting for specific materials, systems and methods

We require information from authors about some types of materials, experimental systems and methods used in many studies. Here, indicate whether each material, system or method listed is relevant to your study. If you are not sure if a list item applies to your research, read the appropriate section before selecting a response.

Materials & experimental systems

n/a	Involved in the study
☒	☐ Antibodies
☒	☐ Eukaryotic cell lines
☒	☐ Palaeontology and archaeology
☒	☐ Animals and other organisms
☒	☐ Clinical data
☒	☐ Dual use research of concern

Methods

n/a	Involved in the study
☒	☐ ChIP-seq
☒	☐ Flow cytometry
☒	☐ MRI-based neuroimaging
